# The prevalence of hypertension in paediatric Turner syndrome: a systematic review and meta-analysis

**DOI:** 10.1038/s41371-022-00777-8

**Published:** 2022-12-05

**Authors:** Sarah McCarrison, Aoife Carr, Sze Choong Wong, Avril Mason

**Affiliations:** 1 Department of Paediatric Endocrinology, Royal Hospital for Children, Glasgow, UK; 2 School of Medicine, University of Glasgow, Glasgow, UK

**Keywords:** Hypertension, Endocrine system and metabolic diseases

## Abstract

Cardiovascular related deaths account for over 40% of the excess mortality in Turner syndrome (TS). Hypertension, a modifiable risk factor for both aortic dilatation and dissection, is more commonly encountered in TS during childhood and adolescence. Treatment of hypertension is currently recommended beyond the age of 16 years in TS to help reduce the risk of aortic dissection. This study aims to determine the prevalence of hypertension in paediatric patients with TS and explore the associated methodologies of blood pressure evaluation reported in these studies. Three online databases were searched (Medline, Embase and Web of Science) for literature which reported a prevalence, or allowed calculation of prevalence, of hypertension in patients with TS who were 18 years of age or younger. Seventeen studies which met the primary eligibility criteria, with a total of 1948 patients, were included. The estimated pooled prevalence of hypertension in children and adolescents with TS was 16% (95% CI: 8.9–24.6%). There was significant heterogeneity detected between the studies. The prevalence of hypertension in those studies which assessed 24-h Ambulatory Blood Pressure Monitoring (ABPM) was 21.1% (95% CI: 15.2–27.6%) compared those which used another method of blood pressure measurement which was 13.5% (95% CI: 5.2–24.4%). Given the impact of hypertension with long-term health outcomes and the reversibility of these same outcomes by addressing abnormal blood pressure, prompt and early diagnosis of hypertension in young girls with TS should be prioritised. We recommend the use of 24-h ABPM in screening for hypertension in the paediatric TS population.

## Introduction

Turner syndrome (TS) is a chromosomal disorder characterised by an abnormality of partial or complete absence of one of the X chromosomes in a phenotypic female. It affects ~1 in every 2000–2500 live born females. Whilst short stature and primary ovarian failure are characteristics of TS, a variety of health issues can present throughout the lifespan of a person with TS.

Congenital cardiac anomalies are present in ~50% of girls with TS, of which bicuspid aortic valve (20–30%), and coarctation of the aorta (4–12%), are most frequently reported [1, 2]. Aortic dilatation and aortic dissection can be attributed to the excess mortality seen in women with TS. The risk of aortic dissection in women with TS is up to 100-fold higher compared to the general population and is associated with a significantly high risk of mortality [3]. Aortic dissection in TS occurs at a much younger age in the second and third decade compared to non-TS related aortic dissection [4]. Whilst congenital anomalies in particular bicuspid aortic valves contribute to this excess risk of aortic dissection, non-congenital circulatory disease is also thought to play a role [5, 6]. Hypertension is often observed in girls and women with TS which is an important factor contributing to aortic dilatation, leading to aortic dissection [7]. Studies in adults with TS document a frequency of hypertension of up to 50% [8]. Of concern is that published research reports a frequency of hypertension in children and adolescents with TS of up to 40% [9–11] However, published studies in growing children with TS use a variety of different methods of assessment of blood pressure; and with many studies of small sample size.

The 2016 international clinical practice guideline for management of TS recommend annual monitoring of blood pressure and initiation of anti-hypertensives if hypertension is diagnosed in girls aged 16 years or older [9]. Clarifying the prevalence of hypertension in children and adolescents with TS is important to guide development of clinical pathways on monitoring and management of this important health issue in children and adolescents with TS. Both European and American paediatric guidelines (non-TS specific) recommend ambulatory blood pressure measurement (ABPM) before starting anti-hypertensive treatment in children [12]. The method of assessment for diagnosis of hypertension in children and adolescents with TS was not specified in the international clinical practice guideline for TS [9]. Therefore, when assessing the prevalence of hypertension in a paediatric population, it is also important to consider the method of blood pressure measurement.

The objective of this systematic review and meta-analysis is to determine the prevalence of hypertension in paediatric patients with TS who are 18 years of age or younger and explore the associated methodologies of blood pressure evaluation reported in these studies on the prevalence.

## Methods

The systematic review of the literature was performed per the Preferred Reporting Items for Systematic Reviews and Meta-analysis guidelines [13].

### Eligibility criteria

All studies which reported a prevalence or allowed calculation of prevalence of hypertension in children and adolescents with TS who were 18 years of age or younger were included in this review. We included studies reporting on all methods of blood pressure assessment and all definitions of hypertension which fulfilled the primary eligibility criteria. In papers which included adult subjects >18 years, if group data for subjects ≤18 years was able to be evaluated independently, this was included in our review. In longitudinal studies or studies which assessed any treatment in TS, only baseline blood pressure and hypertension data was used for purposes of our analysis. Papers that only provided group comparison of blood pressure to a control population were excluded. No publication date restrictions were imposed. Case reports, unpublished manuscripts, review articles and conference abstracts were excluded. Only studies reported in the English language were included in this review.

### Information sources, search strategy and selection process

Two researchers (SM and AC) independently carried out the literature search in May 2021. Studies were identified by searching three online databases: Medline, Embase and Web of Science. Medline and Embase were searched through the Ovid interface. Broad search terms were used, which included the key terms ‘Turner Syndrome’ or ‘Turners’ and ‘Hypertension’ or ‘Blood pressure’. A decision was made not to include the terms ‘Paediatric’ or ‘Children’ in the search strategy to achieve a comprehensive search. Full details of the searches carried out can be found in the Supplementary Information provided.

Duplicates identified during the initial search were first removed. The title, abstract and keywords of the remaining studies were then independently screened by the two researchers (SM and AC). In addition, reference lists of relevant review articles identified were manually searched for any papers which met the eligibility criteria. After the potentially relevant studies were identified, full-text records of these studies were sought and then independently reviewed. All studies which then met the eligibility criteria above were included. Any disagreements were reviewed by a third researcher (AM) to reach a consensus.

### Data collection progress and data items

A standardised data extraction proforma was designed and the relevant data was extracted independently from half the included studies by SM and the other half by AC. The extracted data were then crossed checked by the other reviewer to confirm accuracy of the data. The collected data included the title and authors of the study, year of publication, study design, age of subjects, sample size, method of blood pressure measurement, definition of hypertension and prevalence of hypertension. If more than one method of blood pressure assessment was included in the study, the prevalence data of hypertension were sought and reported for each method.

### Study risk of bias assessment

A risk of bias tool designed for systematic reviews of prevalence studies was used [14]. The tool included ten domains which assessed both external and internal validity. We also provided a numerical score of the risk of bias based on an adapted tool as described by Ciona et al. [15]. A numerical score of ‘1’ was given if risk of bias was deemed low or ‘0’ if the risk was deemed high. A maximal numerical score out of 10 could be assigned for each paper which then classified the overall risk of study bias as low (>8), moderate [6–8] or high (≤5). If there was insufficient information in a paper to permit judgement for a particular domain, a score of ‘0’ was attributed to that item.

External validity items included: assessment of study’s target population, the sampling frame, assessment of random selection and non-response bias. Internal validity items included: Data collection (was the same mode used for all subjects and was the data collected directly from the subjects), assessment of case definition, the reliability and validity of the study instrument, the prevalence period and the appropriateness of numerator and denominator of the parameter of interest.

The risk of bias assessment was carried out independently by two researchers (SM and AC). Any disagreements were reviewed by a third researcher (AM) to reach a consensus.

### Statistics

The primary outcome of this review was the pooled prevalence of hypertension in children and adolescents TS 18 years or younger. For each included study, the prevalence of hypertension stated within the study was included in the meta-analysis. For studies that reported more than one prevalence as more than one method of assessment of blood pressure was used in the same group of subjects, only one prevalence was included in the meta-analysis from that study.

For those studies, we selected the method of blood pressure measurement in the following preferential order: (1) 24-h ABPM, (2) Mercury sphygmomanometer, (3) Oscillometric device, (4) Data collection from clinical records, (5) Self-reported questionnaire.

Meta-analysis was conducted using a random-effects model. As some studies reported extremely low prevalence, we applied the Freeman-Tukey double arscine variance stabilising transformation to the raw prevalence data. The transformed summary proportions were then converted back to proportion estimates for reporting. These were then reported as prevalence percentages with 95% confidence intervals. The between-study variance heterogeneity of effect size estimates across the studies was assessed using the *Q*-test and the *I*^2^ statistic. It was assumed that an *I*^2^ of 25%, 50%, and 75% indicates low, medium, and large heterogeneity, respectively. Visual inspection of a forest plot was also carried out as well as identifying any outliers using leave-one-out diagnostic tests. Subgroup analysis was carried out using a mixed-effects model, by first fitting two separate random-effects models within each subgroup and then combining the estimated statistics from each model using a fixed-effect model to compare the two transformed proportions. A funnel plot was used to investigate publication bias with visual inspection and the Egger test to assess plot asymmetry. All analyses were performed using R Statistical Software (v4.1.3; R Core Team 2022) using the metafor (v3.0-2) and meta (v5.2-0) packages [16–18].

## Results

### Study selection

One thousand three hundred and seventy-four studies were identified through our initial searches. Eight hundred and fifty-one remaining studies were then screened using the title, abstract and keywords. A total of 127 full texts were reviewed to assess eligibility criteria. Forty of these were conference abstracts which were excluded.

**Fig. 1:**
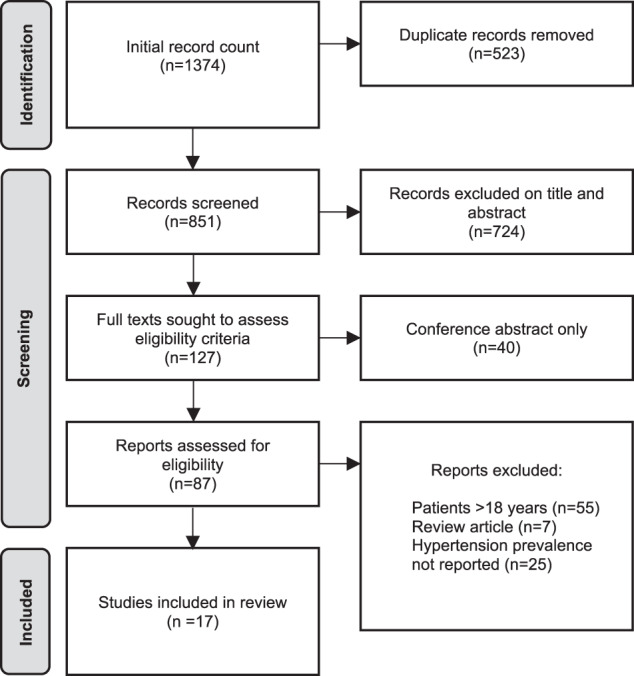
Study selection flowchart. Flowchart detailing study selection process as per the Preferred Reporting Items for Systematic Reviews and Meta-analysis (PRISMA) guidelines. *n* = Number of records.

After reviewing the remaining 87 full texts, 17 studies were included which met primary eligibility criteria. The main reason for exclusion was the inclusion of TS patients >18 years of age or no data to allow for evaluation of prevalence of hypertension in TS. A flowchart detailing this selection process is shown in Fig. 1.

### Study characteristics

A total of 1948 children and adolescents with TS were included in the final 17 studies. Sample size in the included studies ranged from 15 to 842 subjects. The included studies were published between 1992–2020. A detailed summary of study characteristics is included in Table 1.

**Table 1:** Characteristics of the studies included in review.

Author	Year of publication	Study design	Age of study participants (years)	Sample size	Method of BP measurement	Definition of hypertension	Cases	Prevalence (%)	CI (%)
Akyürek et al. [25]	2014	Prospective case-control study	9–17 (Range) 12.4 ± 3.3 (Mean ± SD)	29	1. Mercury sphygmomanometer Additional details given: • Measured 3 times at 1-min intervals. • Subject had rested for at least 10 min. • BP was calculated as the mean of the three measurements. 2. 24-h ABPM Additional details given: • Spacelabs 90207 ambulatory monitor (Spacelabs Medical, Issaquah, WA) used.	1. Clinical BP ≥ the 95th percentile for age, sex, and height. 2. Average SBP or DBP exceeded the 95th percentile for sex and height according to normative values for ABP. Percent dipping was calculated for both average SBP and DBP.	8/29 4/29	27.6 13.8	12.7–47.2 3.9–31.7
Quezada et al. [31]	2015	Prospective cross-sectional study	<18^a^	190	Oscillometric device Additional details given: • DINAMAP 1, GE HealthCare used.	BP percentile score >95 based on previously described data.	57/190	30.0	23.6–37.1
Hamberis et al. [23]	2020	Retrospective cohort study	8.7 (Mean) 4.9–12.4 (IQR)	213	Online health records with ICD-9/ICD-10 diagnoses noted	Not stated	21/213	9.9	6.2–14.7
Tahhan et al. [32]	2019	Prospective cross-sectional study	(Range) 15 ± 2.4 (Mean ± SD)	19	24-h ABPM Additional details given: • SpaceLabs 90217 brand machine used with an adapted paediatric cuff. • Individuals were asked to complete the activity log as instructed by the clinician.	Arterial pressure was considered abnormally high if the 24-h mean arterial pressure exceeded the upper limit of the standard values in >30% of ABPM measurements. The standard values of 1997 ABPM were used in this study. Non-dippers were defined as having <10% nocturnal BP reduction.	5/17)^b^	29.4	10.3–55.9
An et al. [26]	2017	Prospective case-control study	14.6 ± 3.4 (Mean ± SD)	25	Oscillometric device Additional details given: • Colin BP-S510 patient monitor (DRE, Louisville, KY, USA) used. • Patient had relaxed for at least 5 min. • Average of 2 measurements of both arms in the sitting position. • The arm was supported at the heart level. • The cuff bladder encircled at least 80 % of the arm circumference.	Average SBP or DBP ≥95th percentile for sex, age, and height based on the data of National High Blood Pressure Education Program Working Group on High Blood Pressure in Children and Adolescents.	11/25	44.0	24.4–65.1
Fudge et al. [33]	2014	Prospective cross-sectional study	7.1–16.7 (Range)	21^c^	1. Oscillometric device and mercury sphygmomanometer. Additional details given: • Dynamap device (Critikon, Tampa, Fla., USA) and manual mercury sphygmomanometer. • Obtained at the end of the clinic visit. • Phase 1 of the Korotkoff sounds (onset of tapping sounds) corresponded to the systolic BP, and the diastolic BP was determined by phase 5 (the disappearance of tapping sounds) unless Korotkoff sounds could be heard to very low pressures. In these cases, BP measurement was repeated with less pressure on head of stethoscope. Only if Korotkoff sounds persisted at very low pressures, Korotkoff phase 4 was used (muffling of sounds). • An average of 3 readings was used. • Appropriately sized BP cuff (one which extended completely around the circumference of the upper arm with a bladder width that covered at least two thirds of the upper arm) was used. 2. 24-h ABPM Additional details given: • (Spacelabs 90217, Issaquah, Wash., USA) device used. • Placed on a weekday and worn for 24 h while the subjects continued normal activities. They were advised to avoid vigorous exercise including contact sports. • An acoustic signal before recordings during the day reminded the subject to relax the arm. During the night this signal was programmed to be off so as not to interrupt the sleep. • Each subject completed an activity log to record events during the 24-h period which could affect BP (period of physical activity, rest, etc.). • BP recordings were obtained every 20 min during the daytime (08:00–22:00 h) and every 60 min at night (22:00–08:00 h).	1. SBP or DBP >95th percentile for sex, age and height based on normative data for manual BP measurements defined in the 4th report on BP by the National High Blood Pressure Education Program. 2. Mean ambulatory SBP >95th percentile and systolic BP load 25–50% using the criteria suggested by the AHA adapted from Wühl et al. using normative data for ABPM in children stratified by sex and height.	1. 1/21 2. 5/21	4.8 23.8	0.1–23.8 8.2–47.2
De Groote et al. [27]	2017	Prospective case-control study	10.64 (Median) 8.31–11.04 (IQR)	15	1. Oscillometric device Additional details given: • BP taken at the 4 limbs, with the patient at rest and in a supine position. • (Accutorr Plus, Datascope, Mahwah, NJ, USA) used with an appropriate paediatric cuff. • BP was taken 3 times at the right arm during the ultrasound examination. • The mean of these 3 measurements was used for statistics. 2. 24-h ABPM Additional details given: • (Tonoport V, Par Medzintechnik, Berlin, Germany) device used.	1.BP was considered normal if below the 95th percentile for sex, age, and height. 2. Ambulatory BP was analysed using the normal values for sex and age described by Wuhl et al. Abnormal nocturnal dipping was defined.	1. 0/15 2. 4/11	0.0 36.4	0.0–21.8 10.9–69.2
Los et al. [34]	2016	Prospective cross-sectional study	(Range)	168	1. Standardised questionnaire Additional details given: • Self-reported history of a diagnosis of hypertension. 2. Oscillometric device Additional details given: • A single screening BP was measured after a short period of rest (3–5 min) in seated position on the right upper arm. • (Dynamap, GE Healthcare) device used. • Method consistent with American Heart Association recommendations. • Taking the BP on the right arm minimised the possibility of or erroneous measurement in subjects who had previous aortic coarctation operations.	BP ≥95th% was considered evidence of potential hypertension (consistent with definition of stage 1 hypertension or greater).	1. 13/168 2. 71/168	7.7 42.3	4.2–12.9 34.7–50.1
Price et al. [20]	1993	Prospective cohort study	8.7 ± 3.2 (Mean ± SD)^d^	47	Mercury sphygmomanometer Additional details given: • Diastolic BP was determined at Korokoff phase IV. • SBP + DBP was converted to SDS using north American standards.	Not stated	2/47^d^	4.3	0.5–14.5
Zhang et al. [30]	2019	Prospective case-control study	5–18 (Range) 12.22 ± 3.98 (Mean ± SD)	64	Method of blood pressure measurement not stated	SBP or DBP ≥95th percentile for age and sex.	4/64	6.2	1.7–15.2
Sas et al. [35]	1999	Multicentre randomised dose-response study	2–11^d^ (Range)	68	Method of blood pressure measurement not stated Additional details given: • BP was determined with each girl in a sitting position and by using a cuff size corresponding to the size of her arm. • The second to fourth readings were used to calculate the mean systolic and diastolic BP. • BP was expressed as SD score, with age and sex-specific reference values.	Not stated	0/68^d^	0.0	0.0–5.3
Lebenthal et al. [24]	2018	Longitudinal retrospective cohort study	Childhood: 45*,*X 9. 2 ± 1.0 (Mean ± SD) Other karyotypes 9.1 ± 1.4 (Mean ± SD) Adolescence: 45*,*X 13.5 ± 1.3 (Mean ± SD) Other karyotypes 13.7 ± 1.2 (Mean ± SD)	Childhood: 87 Adolescence: 91	Method of blood pressure measurement not stated Additional details given: • Blood pressure was measured according to the recommendations of the National High Blood Pressure Education Program (NHBPEP). • In childhood, percentiles for SBP and DBP were calculated according to height, sex, and age.	Defined according to the NHBPEP: hypertension defined when either SBP or DBP ≥95th percentile.	Childhood: 3/87 Adolescence 19/91	Childhood: 3.4 Adolescence 20.9	0.7–9.7 13.1–30.7
Akyürek et al. [28]	2015	Prospective case-control study	9–17 (Range) 12.5 ± 3.30 (Mean ± SD)	29	Mercury sphygmomanometer Additional details given: • Measured 3 times at 1-min intervals. • Subject had rested for at least 10 min. • BP was calculated as the mean of the three measurements.	Clinical BP ≥ the 95th percentile for age, sex, and height.	6/29	20.6	8.0–39.7
McCarthy et al. [19]	2008	Prospective cohort study	7–17 (Range) 12.5 (Mean) 13 (Median)	100	24-h ABPM	Hypertension was determined by ambulatory blood pressure monitoring using height-based standards.	20/100	20.0	12.7–29.2
Lopez et al. [29]	2008	Prospective case-control study	<18 10.2 (Mean)	138	1. Standardised questionnaire Additional details given: • Each subject with Turner syndrome or parent completed a questionnaire regarding karyotype, heart disease, cardiac surgery, hypertension, history of GH therapy, other medications and other medical conditions. 2. Method of blood pressure measurement not stated	Average SBP or DBP that is ≥95th percentile for sex, age, and height using guidelines from the National High Blood Pressure Education Program.	1. 6/138 2. 56/138	4.4% 40.6%	1.6–9.2 32.3–49.3
Valencia et al. [21]	2011	Retrospective cohort study	8–18 years^e^	21	Evaluation of clinical records	Not stated	1/20^e^	5.0	0.1–24.9
Yeşilkaya et al. [22]	2015	Retrospective cohort study	0–18 years	842	Data collection from patient hospital records using a common case recording form	Not stated	15/719^f^	2.1	0.1–3.4

*SD* standard deviation, *IQR* interquartile range, *BP* blood pressure, *SBP* systolic blood pressure, *DBP* diastolic blood pressure, *ABPM* ambulatory blood pressure monitoring, *SDS* standard deviation score.

^a^Further data including mean, SD, median and IQR provided for whole group (3–70 years) but not provided for <18 year population only.

^b^ABPM only performed in 17/19 subjects.

^c^Data extracted from 21/23 subjects ≤18 years included in study.

^d^Baseline data only.

^e^Data extracted from 20/21 subjects ≤18 years included in study.

^f^Cardiovascular data provided for 719/842 subjects.

Two out of 17 (11.8%) studies were prospective longitudinal cohort studies [19, 20], four (23.5%) were retrospective cohort studies of clinical evaluation [21–24], six were prospective case-control studies (35.3%) [25–30], four (23.5%) were prospective cross-sectional studies [31–34] and one (5.9%) was a multicentre randomised dose-response study of growth hormone treatment [35].

Five (29.4%) out of the studies used more than one method of assessment of blood pressure [25, 27, 29, 33, 34]. Five studies reported 24-h ABPM [19, 25, 27, 32, 33]. Four studies used a mercury sphygmomanometer as the method of assessment of blood pressure [20, 25, 28, 33]. Five used an oscillometric device [26, 27, 31, 33, 34]. Three studies used data from clinical records [21–23] and two used questionnaires in their assessment [29, 34]. Three studies did not state the method of blood pressure assessment in their methodology [24, 30, 35]. Of the five studies which used more than one method of blood pressure assessment, one study reported only one out of two of their methods of assessment [29].

Five studies did not report the definition of hypertension used in their methodology [20–23, 35]. In those which assessed ABPM, three studies used a definition of mean ambulatory BP > 95^th^ centile based on AHA recommendations using normative data adapted from Wuhl et al. [25, 27, 33, 36, 37]. One study defined hypertension if the 24-h mean BP exceeded the upper limit of the standard values in >30% of ABPM measurements using 1997 ABPM standard values [32, 38]. One study reported hypertension as determined by height-based standards of ABPM but gave no further detail on this [19]. The remaining studies reported BP assessment using mercury sphygmomanometer, oscillometric device or an undefined method used a definition of hypertension as ≥ the 95th percentile for age, sex, and height [24–31, 33, 34].

### Risk of bias in studies

A risk of bias assessment for each included study is shown in Table 2. All included studies were classified to be at moderate (76.5%) or high (23.5%) risk of bias based on the assessment of internal and external validity items. No study included in our review reported a form of random selection or census used to select the sample or provided an analysis comparing responders and non-responders. In three studies, it could not be determined whether blood pressure was collected directly from the subjects as measurements were taken from online health records [21–23]. Five studies provided no case definition for hypertension [20–23, 35] and one study was deemed to have an unacceptable case definition with not enough information provided [19]. Eight studies either did not specify the instrument used for blood pressure assessment or did not provide sufficient information for assessment of reliability and validity of the method blood pressure assessment [21–24, 29–31, 35].

**Table 2:** Risk of bias assessment of included studies.

Author (Year of Publication)	External validity items				Internal validity items						Overall score	Overall risk of bias
	1	2	3	4	5	6	7	8	9	10		
Akyürek (2014) [25]	1	1	0	0	1	1	1	1	1	1	8	Moderate
Quezada (2015) [31]	1	1	0	0	1	1	0	1	1	1	7	Moderate
Hamberis (2020) [23]	1	0	0	0	0	0	0	0	1	1	3	High
Tahhan (2019) [32]	1	1	0	0	1	1	1	1	1	1	8	Moderate
An (2017) [26]	1	1	0	0	1	1	1	1	1	1	8	Moderate
Fudge (2014) [33]	1	1	0	0	1	1	1	1	1	1	8	Moderate
De Groote (2017) [27]	1	1	0	0	1	1	1	1	1	1	8	Moderate
Los (2016) [34]	1	1	0	0	1	1	1	1	1	1	8	Moderate
Price (1993) [20]	1	0	0	0	1	0	1	1	1	1	6	Moderate
Zhang (2019) [30]	1	1	0	0	1	1	0	0	1	1	6	Moderate
Sas (1999) [35]	1	0	0	0	1	0	0	0	1	1	4	High
Lebenthal (2018) [24]	1	1	0	0	1	1	0	0	1	1	6	Moderate
Akyürek (2015) [28]	1	1	0	0	1	1	1	1	1	1	8	Moderate
McCarthy (2008) [19]	1	1	0	0	1	0	1	1	1	1	7	Moderate
Lopez (2008) [29]	1	1	0	0	1	1	0	0	1	1	6	Moderate
Valencia (2011) [21]	1	1	0	0	0	0	0	0	1	1	4	High
Yeşilkaya (2015) [22]	1	1	0	0	0	0	0	0	1	1	4	High

Items scored: 1. Was the study’s target population a close representation of the national population in relation to relevant variables? 2. Was the sampling frame a true or close representation of the target population? 3. Was some form of random selection used to select the sample, OR, was a census undertaken? 4. Was the likelihood of non-response bias minimal? 5. Were data collected directly from the subjects? 6. Was an acceptable case definition used in the study? 7. Had the study instrument that measured the parameter of interest been tested for reliability and validity? 8. Was the same mode of data collection used for all subjects? 9. Was the length of the shortest prevalence period for the parameter of interest appropriate? 10. Were the numerator(s) and denominator(s) for the parameter of interest appropriate? Answers: 0 = No, 1 = Yes. Overall risk of bias: Low (score > 8), moderate (score 6–8), or high (score ≤ 5).

### Results of individual studies

The overall prevalence of hypertension of the studies reviewed ranged from 0–44%. The prevalence of hypertension with the associated 95% confidence interval for each individual study is shown in Table 1. In those studies which assessed 24-h ABPM, the prevalence ranged from 13.8–36.4%. In those which used a mercury sphygmomanometer, the prevalence ranged from 4.3–27.6%. Prevalence in studies which used an oscillometric device was 0–44%.

### Pooled prevalence estimate

**Fig. 2:**
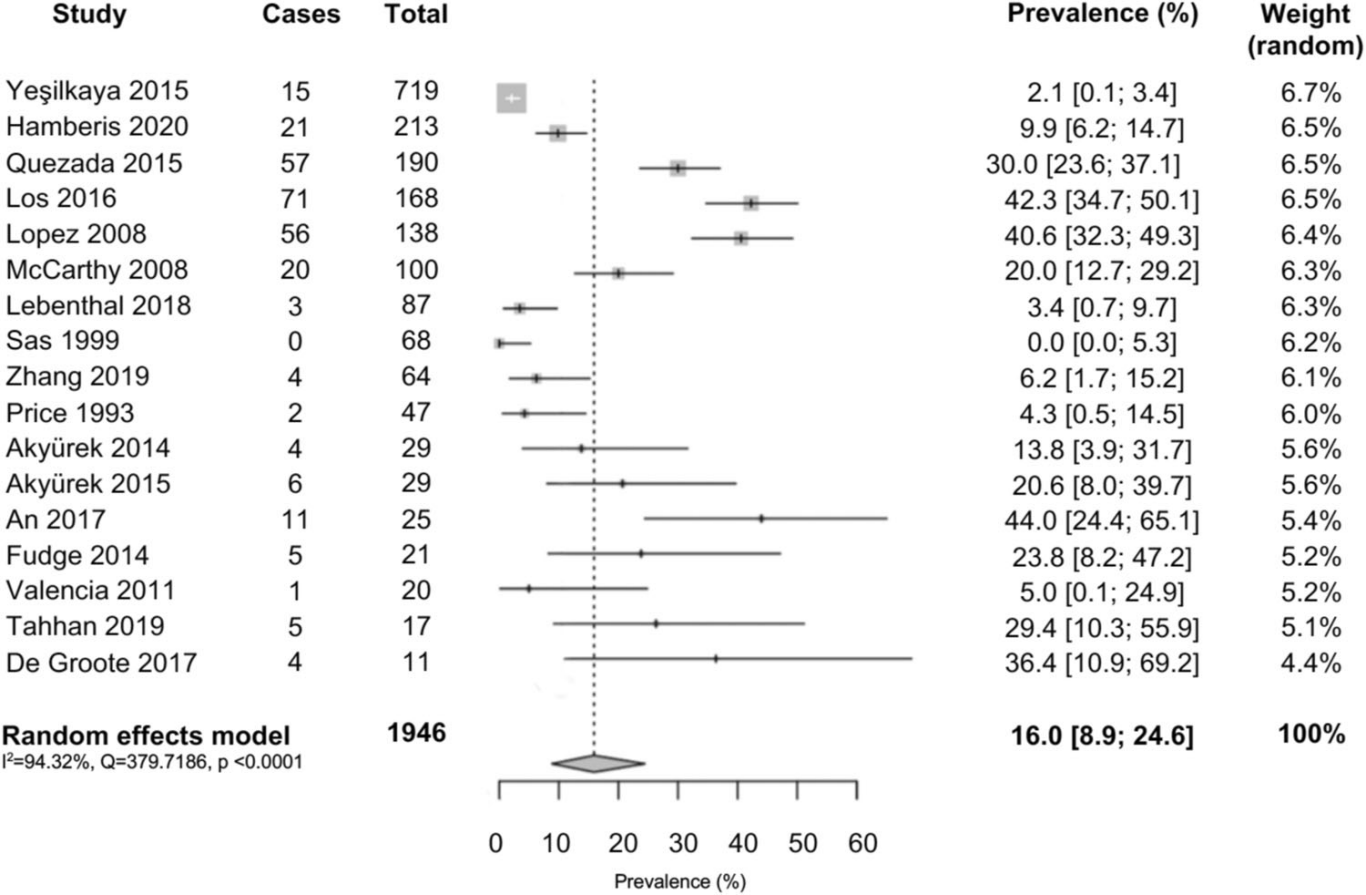
Forest plot of hypertension in paediatric Turner syndrome. Results of meta-analysis using random-effects model are shown with the estimated pooled prevalence of hypertension in paediatric Turner syndrome along with 95% confidence intervals. *I*^2^ was 94.32% and Q-test for heterogeneity was statistically significant at 379.7 (*p* < 0.0001).

**Fig. 3:**
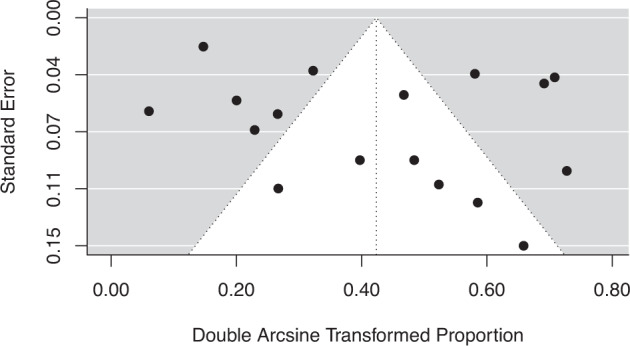
Funnel plot showing estimated prevalence of hypertension in paediatric Turner syndrome using transformed proportions. Each dot represents a separate study. The double-arscine transformed proportion of children and adolescents with Turner syndrome <18 years with hypertension relative to the total sample size (as a measure of prevalence) in each individual study on the *x*-axis plotted against its standard error (as a measure of study precision) on the *y*-axis. Visual inspection suggested no asymmetry.

The estimated pooled prevalence of hypertension in children and adolescents with TS using a random-effects model was 16% (95% CI: 8.9–24.6%). This is shown in the Forest plot in Fig. 2. There was significant heterogeneity detected between the studies. *I*^2^ was 94.32% and *Q*-test for heterogeneity was statistically significant at 379.7 (*p* < 0.0001). A visual inspection of summary proportions leaving out a study at a time revealed no outliers. This was confirmed with a series of leave-1-out diagnostic tests which revealed no influential studies. Funnel plot shown in Fig. 3 demonstrated no asymmetry and *p* value for Egger’s test was not significant (*p* = 0.3123), suggesting no publication bias was detected.

### Subgroup analysis

Subgroup analysis was performed due to significant heterogeneity detected between the studies. This is shown in Table 3. There was a noteworthy difference between subgroups in those studies with a high risk of bias compared with those with a moderate risk of bias. No differences were noted with method of blood pressure assessment or year of publication.

**Table 3:** Results of subgroup analysis.

Subgroup	Number of studies	Total number of subjects	Cases	*I*^2^ (%)	Prevalence (%)	95% CI (%)	Subgroup difference (*p* value)
Method of blood pressure measurement							
24-h ABPM	5	178	38	0	21.1	15.2–27.6	0.217
Others	12	1768	247	96.4	13.5	5.2–24.4	
Risk of bias							
Moderate	13	926	248	89.1	21.9	12.7–31.4	0.002
High	4	1020	37	88.1	2.7	0.0–8.6	
Study year							
After 2012	12	1573	206	94.0	18.4	9.9–28.7	0.405
Before 2012	5	373	79	94.2	10.7	0.6–28.4	

*ABPM* ambulatory blood pressure monitoring.

According to method of blood pressure measurement, the prevalence of hypertension in those studies which assessed 24-h ABPM was 21.1% (15.2–27.6). Those which used another method of blood pressure measurement, detailed above, showed a prevalence of 13.5% (5.2–24.4%). According to the risk of bias noted in the studies, the prevalence of hypertension in those studies with a moderate risk of bias was 21.9% (95% CI: 12.7–31.4%). Those with a high risk of bias showed a prevalence of 2.7% (95% CI 0.9–8.6%). This difference was noted to be statistically significant (*p* = 0.002). The prevalence of hypertension in studies carried out in the last 10 years (after 2012) was 18.4% (95% CI: 9.9–28.7%), compared with those before 2012 which showed a prevalence of 10.7% (95% CI: 0.6–28.4%).

## Discussion

This systematic review and meta-analysis showed a 16% prevalence of hypertension in children and adolescents with TS and is notably higher than estimates of childhood hypertension world-wide, thought to be ~4% [39]. Our subgroup analysis identified a prevalence of hypertension of 21.2% in TS in studies which used 24-h ABPM as the method of assessment of blood pressure. Previous studies which have compared blood pressure in children with TS to a control population have also shown higher blood pressures in these girls from a young age [40]. Therefore, greater focus should now be given on early accurate identification and management of hypertension in children and adolescents with TS.

The underlying pathogenesis of hypertension in TS is multifactorial with impaired vagal tone, congenital cardiovascular and renal anomalies, higher cortisol levels, impaired insulin resistance and the metabolic syndrome in the context of obesity all considered to be potential contributing factors [41]. In addition, oestrogen deficiency is felt to contribute in part to this and studies in adult patients have shown a beneficial effect on blood pressure with sex hormone replacement [42, 43]. However, a recent study in young adults with TS showed that despite effective oestrogen replacement, blood pressure increased significantly during adolescence and young adulthood [44]. The cause of this increase in blood pressure early in life in TS is still unknown.

Not only has hypertension been identified as a risk factor for aortic dissection in TS but the end-organ effects of hypertension including cardiovascular disease, cerebrovascular damage and nephropathy are well documented in the general population [45]. Children with TS have also been shown to have additional risk factors for cardiovascular morbidity with vasculopathy detected as early as 9 years of age [46]. Thus, further research in understanding the underlying aetiology of hypertension in young girls with TS may help in the development of effective management strategies.

It is well known that practical difficulties in clinic blood pressure measurement in children can lead to both under- and over- estimation of blood pressure readings [47]. Due to these difficulties, 24-h ABPM gives a more accurate representation of assessment of hypertension in a paediatric population [48]. This is also supported by two studies in our review who identified girls with hypertension with 24-h ABPM that were not picked up by resting BP alone [27, 33]. Our subgroup analysis shows that the pick-up with 24-h ABPM may be higher, with the prevalence of hypertension in those studies that assessed 24-h ABPM being 21.1% compared to 13.5% in those studies which did not assess 24-h ABPM. Studies which have assessed ABPM in a young TS population have also shown a lack of normal diurnal variation with less than the normal 10% reduction in nocturnal systolic blood pressure in up to 57% of patients [10, 11]. Lack of this normal mechanism, termed ‘non-dipper’, has been associated with increased morbidity and is likely to play a part in the end-organ damage, including left ventricular hypertrophy observed in ‘normotensive’ women with TS [49, 50].

**Fig. 4:**
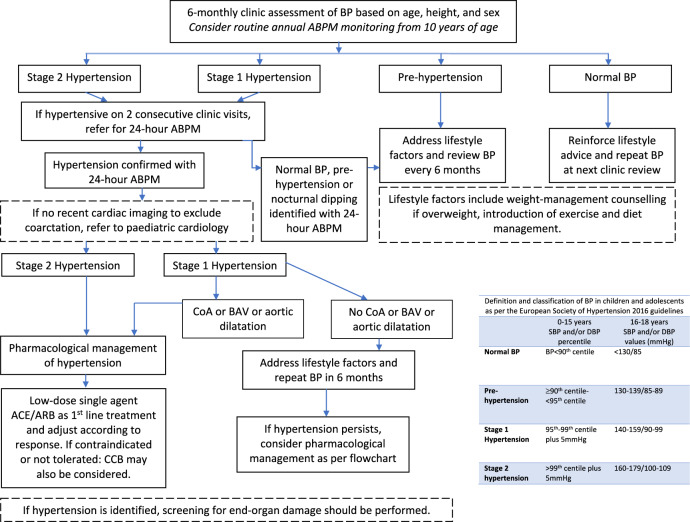
Suggested pathway for identification and management of hypertension in children and adolescents with Turner syndrome <18 years of age. BP blood pressure, ABPM ambulatory blood pressure monitoring, CoA coarctation of the aorta, BAV bicuspid aortic valve, ACE angiotensin-converting enzyme inhibitor, ARB angiotensin receptor blocker, CCB calcium channel blocker.

The 2016 international clinical practice guideline suggests individuals with TS should have monitoring of blood pressure on an annual basis but no further detail on how this monitoring should be carried out is provided, especially for children and adolescents. Anxiety disorders are common in girls with TS [51]; and clinic measured blood pressure may not allow differentiation between true hypertension and white coat hypertension. Studies which have assessed the natural history of hypertension in a TS population have shown an increased prevalence of hypertension with age [41, 52]. To the best of our knowledge, there are no published longitudinal studies of blood pressure monitoring using 24-h ABPM in children and adolescents with TS. There is evidence in adults (although not specific to the TS population) that aggressive treatment of blood pressure not only stops but can reverse end organ damage [45]. We therefore suggest that 24-h ABPM should be gold standard for monitoring in adolescents with TS from the age of 10–12 years. Early treatment with anti-hypertensives should be initiated in TS girls (even in those <16 years) identified as hypertensive with 24-h ABPM. In normotensive girls identified through ABPM to be non-dippers, closer monitoring of blood pressure should be initiated. Whilst out of the scope of the current review, we also recommend greater focus on more intensive lifestyle modification (physical activity and nutrition) in these young growing girls with abnormal blood pressure. A suggested pathway for monitoring, diagnosis, and treatment of hypertension in children and adolescents with TS in shown in Fig. 4. There is some evidence that the renin-aldosterone-angiotensin system and sympathetic nervous system appear to be altered in TS and therefore we suggest hypertensive agents targeting these systems may have a beneficial role in treatment of hypertension in these patients [42, 53, 54]. However, we acknowledge there is little existing evidence assessing management of hypertension in children and adolescents and further systematic reviews will be required to explore this further.

Our study has a number of limitations which should be considered. The pooled prevalence of hypertension should be interpreted with some caution, considering the large heterogeneity between the studies. Many of the studies included in our review had small sample sizes and all studies had a moderate to high risk of bias. We identified a number of studies which included subjects up to the age of 22 which were not included in our study due to the age restrictions we set at the beginning of the review process to ensure the results were generalisable to a purely paediatric TS population [10, 11, 55]. Whilst these limitations should be considered, it is important to note that this review presents the current published data on the prevalence of this important health condition in a young TS population.

There is a distinct need for more high-quality observational studies assessing the natural history of hypertension in a paediatric TS population with clear reporting of methodology, definitions, and confounding factors. Such studies are needed to develop evidence based clinical guidelines of monitoring and management in young growing children with TS, with the goal of reducing the risk of long-term adverse cardiovascular outcomes including aortic dissection.

## Conclusion

To our knowledge, this is the first systematic review and meta-analysis evaluating the prevalence of hypertension in a paediatric TS population. Given the impact of hypertension with long-term health outcomes and the reversibility of these same health risks achieved by addressing abnormal blood pressure, prompt and early diagnosis of hypertension in young girls with TS should be prioritised.

## Supplementary information

Supplementary Material

## Data Availability

Data generated or analysed during this study are included in this published article and its Supplementary Information files. Any further data can be made available from the corresponding author on reasonable request.
